# Strategies for recombinant laccase expression and their roles in environmental remediation

**DOI:** 10.1007/s11274-026-04978-y

**Published:** 2026-04-27

**Authors:** Isabeli Bannach-Machado, Rafael Trindade Maia, Rosane Marina Peralta, Cristina Giatti Marques de Souza, Charles Windson Isidoro Haminiuk, Giselle Maria Maciel

**Affiliations:** 1 Graduate Program in Environmental Science and Technology, Federal University of Technology – Paraná (UTFPR), Curitiba, Paraná 81280-340 Brazil; 2 Graduate Program in Natural Sciences and Biotechnology, Federal University of Campina Grande (UFCG), Cuité, Paraíba 58175-000 Brazil; 3 Graduate Program in Cellular and Molecular Biology, Federal University of Paraíba, João Pessoa, Paraíba 58051-900 Brazil; 4 Graduate Program in Biochemistry, State University of Maringá (UEM), Maringá, Paraná 87020-900 Brazil

**Keywords:** Recombinant laccase, Bacterial laccases, Fungal laccases, Environmental applications, Heterologous expression, Protein engineering

## Abstract

Laccases are multicopper enzymes capable of oxidizing a wide variety of compounds, standing out as green tools for industrial and environmental applications. However, production from native sources faces limitations that have driven advances in recombinant expression. This scoping review includes studies published between 2019 and 2025, selected from major online databases, describing recombinant fungal and bacterial laccases for environmental applications. Strategies to optimize expression are discussed, including the use of efficient vectors, codon optimization, His-tag addition, mutagenesis, and computational approaches, with an emphasis on their advantages, trade-offs, and limitations. *Pichia pastoris* is widely used for the expression of fungal laccases, while *Escherichia coli* is preferred for the expression of bacterial laccases. However, significant variability in expression efficiency and enzyme performance is observed across hosts and constructs. The use of alternative culture media, such as agro-industrial residues, is also explored as a sustainability-driven strategy; however, its applicability remains limited for certain heterologous expression systems. In the environmental field, recombinant laccases demonstrate high efficiency in the degradation of textile dyes, the treatment of lignocellulosic waste, the biodegradation of pharmaceuticals, and the degradation of various toxic compounds, often requiring redox mediators to achieve high conversion rates. Despite significant advances, challenges remain, such as inconsistent catalytic performance among studies and limited stability under extreme temperature and pH conditions. Overall, this review highlights the key challenges in developing recombinant laccase and demonstrates that advances in protein engineering, expression systems, and process optimization are crucial for environmental applications.

## Introduction

Laccases (EC 1.10.3.2) are enzymes classified as multicopper oxidoreductases, with protein sequence lengths ranging from 220 to 800 amino acid residues and molecular weights between 50 and 140 kDa (Rahman et al. 2024). These glycoproteins exhibit a remarkable ability to catalyze the oxidation of a wide range of substrates, including phenols, aromatic amines, carboxylic acids, organometallic compounds, and other non-phenolic substrates (Li et al. 2021). Consequently, they stand out for their potential in green catalysis, encompassing applications from the synthesis of organic compounds to the treatment of environmental pollutants (Brugnari et al. 2021).

Moreover, laccases are widely distributed across fungi, bacteria, insects, plants, lichens, and algae (Trubitsina et al. 2021). Most of the laccases characterized to date are of bacterial or fungal origin, primarily due to the relative ease of enzyme purification (Xu et al. 2019). It is worth noting that their function is diverse and intrinsically linked to the nature of the host organism (Mahuri et al. 2023). Although they share similar structural features, laccases exhibit considerable heterogeneity, particularly in their optimal temperature and pH ranges and substrate specificity.

**Fig. 1:**
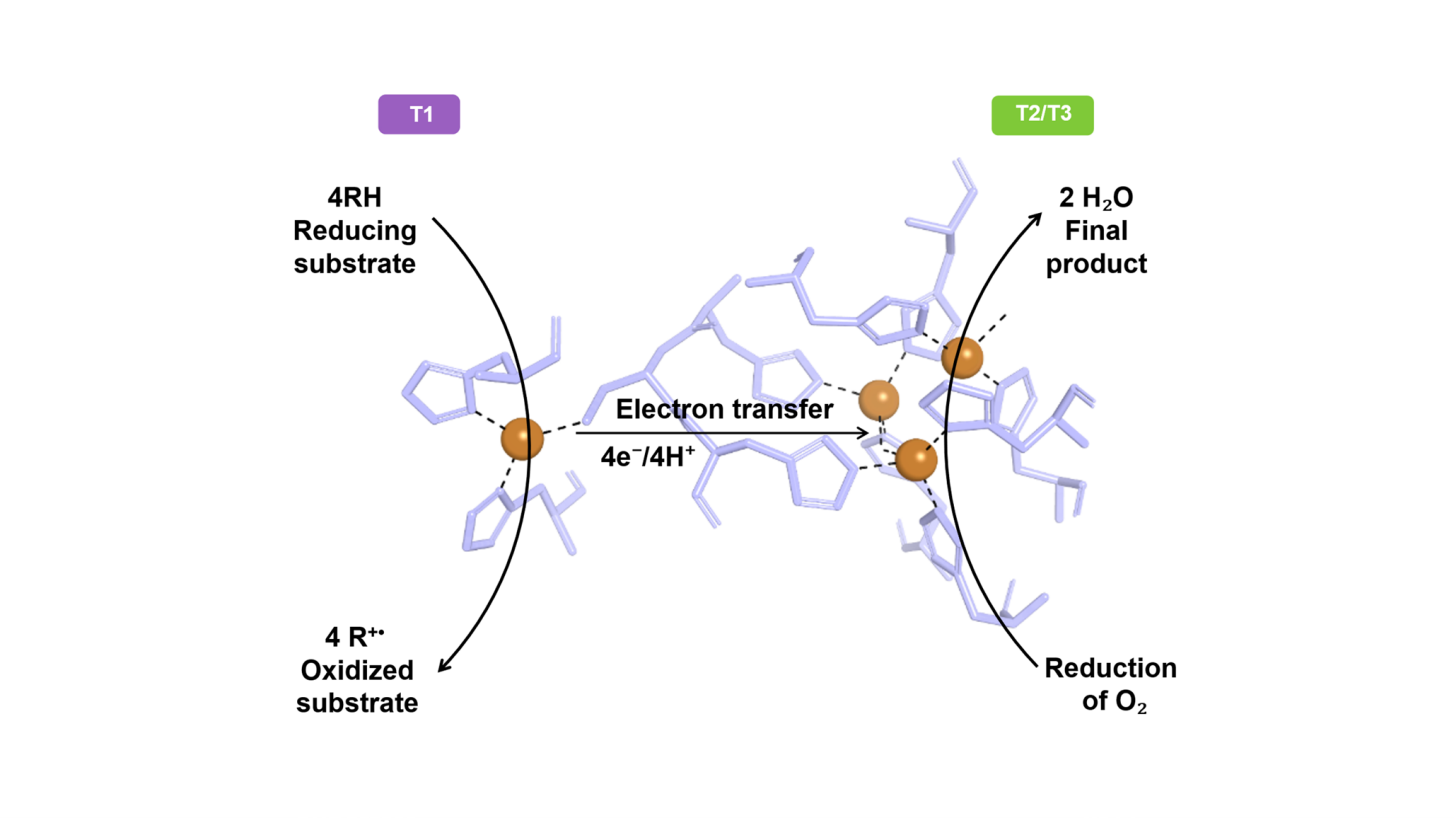
Scheme of catalytic mechanism of laccase (PDB ID: 1GYC). The substrate (RH) is oxidized at the type 1 copper (T1) site, generating radical species (R•) and releasing electrons. These electrons are transferred to the trinuclear copper cluster (T2/T3), where molecular oxygen (O₂) is reduced to water. Residues within 3 Å of the copper centers are shown in light blue, copper ions are represented as gold-colored spheres, and dashed lines indicate coordination interactions

Laccases catalyze the oxidation of substrates through the active participation of copper atoms. The process involves electron transfer, the formation of unstable radicals, and ultimately the reduction of molecular oxygen to water (Wiśniewska et al. 2021) (Fig. 1). Since water is the only byproduct released, laccases are considered among the most environmentally friendly enzymes currently known (Pezzella et al. 2017). Their redox potential typically ranges from + 430 to + 800 mV (vs. the normal hydrogen electrode), directly influencing their ability to oxidize structurally diverse and recalcitrant compounds (Wiśniewska et al. 2021). In this context, fungal laccases generally exhibit higher redox potentials than bacterial counterparts, which partially explains their superior performance toward high-redox-potential substrates (Pezzella et al. 2017).

Fungal laccases are involved in various biological processes, including fungal morphogenesis and lignification/delignification (Niglio et al. 2019; Wang et al. 2024).They are typically active at acidic pH (Kontro et al. 2021) and are generally extracellular and glycosylated (Huy et al. 2021; Zhuo et al. 2019). In contrast, bacterial laccases differ from fungal ones in several respects, including higher activity under neutral to alkaline pH conditions and enhanced tolerance to elevated temperatures, which make them attractive for applications requiring operational robustness (Ahlawat et al. 2019; Kumar et al. 2021; Wang et al. 2019).

Due to their catalytic versatility, laccases have numerous biotechnological applications. The most frequently cited uses of laccases include textile industry applications, dye degradation, environmental pollutant remediation, detoxification of solid waste and composting, biosensors, delignification, food processing, the pulp and paper industry, and polymer synthesis (Brugnari et al. 2021; Younus et al. 2025; Zofair et al. 2022). Despite these promising applications, the large-scale production of laccases from native sources remains challenging. Many laccase-producing microorganisms require specific growth conditions, exhibit slow growth rates, or produce low enzyme titers, resulting in high production costs and limited industrial feasibility (Mahuri et al. 2023; Niglio et al. 2019).

To overcome these limitations, recombinant protein expression has emerged as a key strategy to improve laccase availability and reduce production costs. Heterologous expression in easily cultivable hosts enables higher productivity, shorter fermentation times, and greater process control (Li et al. 2021). Enzyme yields can be further enhanced by using multiple gene copies, optimized signal peptides, and strong promoters (Asemoloye and Marchisio 2022; Cao et al. 2021; Cheng et al. 2021). In addition, protein engineering approaches have been widely applied to improve catalytic efficiency, stability, and/or substrate specificity, allowing the development of tailored laccases for specific applications (Huang et al. 2024; Savinova et al. 2020). As a result, the heterologous expression of laccases genes in bacteria, yeasts, filamentous fungi, and plants has been widely reported (Rahman et al. 2024; Trubitsina et al. 2021).

Thus, the generation of recombinant laccases emerges as a promising strategy to meet the increasing demand for versatile and efficient enzymes in industrial and environmental processes, aligning with the principles of sustainable development and environmentally friendly practices (Niglio et al. 2019). Nevertheless, the production of recombinant laccase faces some limitations. Expression efficiency, post-translational modifications, and catalytic performance vary substantially depending on the host system and engineering strategy employed (Huang et al. 2024; Kumar et al. 2021). Moreover, environmental applications are often limited by mediator dependence and instability under extreme pH and temperature conditions (Ahlawat et al. 2019; Song et al. 2020).

In recent years, several comprehensive reviews have addressed the structure, catalytic mechanisms, and biotechnological applications of laccases, highlighting advances in protein engineering, redox mediators, and their roles in environmental remediation and biocatalysis. These studies emphasize their potential in pollutant degradation and wastewater treatment (Brugnari et al. 2021). Ardila-Leal et al. (2021) offer a unique perspective on the use and removal of natural and synthetic dyes by laccases from a historical and socio-artistic viewpoint. Applications in the food industry, including production from agro-industrial byproducts and bioprocessing, have also been explored (Mayolo-Deloisa et al. 2020). Additionally, recent reviews have examined enzyme immobilization strategies using biomaterials and agro-industrial residues, discussing their advantages and limitations (Alvarado-Ramírez et al. 2021). However, a comprehensive and up-to-date analysis focusing specifically on recombinant laccases is still needed.

In this context, the aim of this article was to review and synthesize the recent advances on recombinant laccases from January 2019 to April 2025, focusing on their characteristics, catalytic efficiency, optimization, and stability. Additionally, this work discusses the sustainable applications of these technologies and the challenges encountered in the environmental field.

## Review criteria

The purpose of this review article was to examine and compile the available knowledge on recombinant laccases from 2019 to April 2025, with an emphasis on their potential for environmental applications and biotechnological advancements. To achieve this, an initial literature search was conducted using the Web of Science and Scopus databases, as they offer broad coverage of scientific publications across various disciplines and are accessible through the institutional academic account.

Accordingly, the following search terms were selected: “recombinant laccase” OR “heterologous laccase production” OR “laccase engineering” OR “recombinant production of laccase” OR “recombinant expression of laccase” OR “heterologous laccase” OR “genetically modified laccase”. Searches were conducted in the title, abstract, and keywords fields.

Inclusion criteria were: (i) studies reporting recombinant laccases of fungal or bacterial origin; (ii) studies with a clear focus on environmental applications; (iii) peer-reviewed original research articles. Exclusion criteria included: (i) studies on native laccases without recombinant expression; (ii) review articles, conference abstracts, editorials, etc.; (iii) duplicates across databases; and (iv) studies without access to full text.

In the initial search, 71 articles were retrieved from the Web of Science database, while 27 articles were identified in Scopus. After the removal of duplicates and an initial screening based on titles, keywords, and abstracts, 60 articles were selected for further analysis, comprising 24 studies on fungal laccases and 36 studies on bacterial laccases. The selected articles were analyzed with respect to reported application, laccase synonyms, enzyme origin, expression host strain, expression vector, optimal temperature, and optimal pH, which supported a comprehensive description in the review. Additional studies identified in the literature were also cited when considered relevant to the discussion and contextualization of the findings.

### Review and discussion

#### Identification and characterization of microbial laccases

Among microbial laccases, fungal enzymes have been the most extensively studied, both because they were the first microbial laccases to be identified (Coria-Oriundo et al. 2021; Li et al. 2021) and due to their enzymatic properties, such as their high redox potential (~ 470–810 mV) and elevated catalytic activity (Liu et al. 2021), in addition to their broad range of applications, especially in the degradation of various substrates (Huang et al. 2024).

However, fungal laccases may be unstable under alkaline or high-temperature conditions (Li et al. 2021), as they are typically active at acidic pH (3.0–6.0). In contrast, recombinant laccases tend to exhibit greater stability and broader operational ranges (Song et al. 2020). However, such enhancements are highly dependent on the specific enzyme variant and expression strategy employed. For example, the recombinant laccase from *Coprinopsis cinerea* demonstrated improved activity and increased stability over a broader pH range, reaching values of 4.5–9.0 (Xu et al. 2019) and 6.5–7.0 (Kontro et al. 2021), and is classified as a thermophilic and alkaliphilic fungal laccase. Similarly, the recombinant laccase from *Madurella mycetomatis* remained active across a wide pH range (2.0–8.0) (Tülek et al. 2021a) Nevertheless, these examples also illustrate that improved pH tolerance does not necessarily correlate with enhanced catalytic efficiency or substrate versatility (Tülek et al. 2021a) underscoring the variability among recombinant systems.

In contrast, low-redox-potential laccases, derived from ascomycetes and bacteria, exhibit an optimal pH between 6.0 and 9.0 (Kontro et al. 2021). Moreover, some bacterial laccases exhibit high thermal and pH stability, making them robust under harsh conditions (Li et al. 2020; Wang et al. 2019). Additionally, their enzymatic activity is less affected by metal ions, salts, alkalis, organic solvents, and other inhibitors than that of fungal laccases (Hajipour et al. 2020; Jiang et al. 2021; Li et al. 2020). Furthermore, they may remain stable under environmental and industrial conditions, even in the presence of ultraviolet light, hydrogen peroxide (H₂O₂), and other chemical agents (Li et al. 2021).

The ability of bacterial laccases to resist denaturation and remain functional at high temperatures can be attributed to their structural conformation. Li et al. (2021) reported that extensive hydrophobic interactions between the three cupredoxin-like domains, connected by external interdomain loops, promote the formation of a compact catalytic core. This structural compactness not only enhances thermostability but also enables bacterial laccases to retain activity in complex industrial matrices, such as textile effluents characterized by elevated temperatures and fluctuating pH values (Li et al. 2021; Rai et al. 2019). Additional factors contributing to thermostability include a high alanine content (Ahlawat et al. 2019), the presence of disulfide bonds, and conserved amino acid residues such as glutamate, glycine, isoleucine, proline, and arginine (Hajipour et al. 2020). Importantly, the identification of these conserved residues provides valuable targets for rational enzyme engineering to improve laccase robustness and process compatibility (Yan et al. 2022). However, the structure–function relationship of bacterial laccases at high temperatures remains unclear, underscoring the need for further research in this area (Li et al. 2022).

**Fig. 2:**
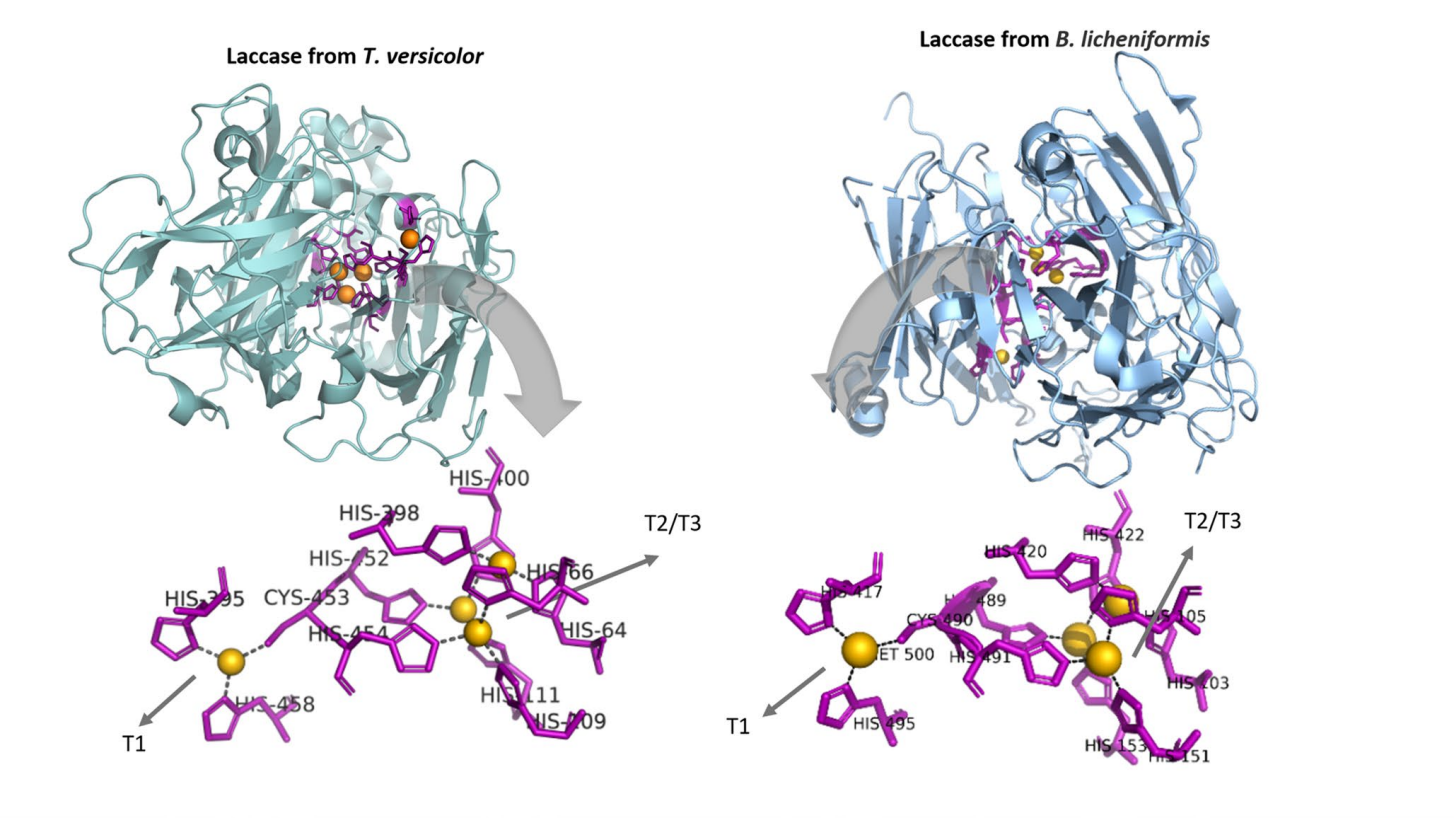
Overall structure and copper centers of laccases from *Trametes versicolor* (PDB code 1GYC) and *Bacillus licheniformis* (PDB code 9BD5). Molecular representations were generated using Pymol

Differences in catalytic efficiency can also be attributed to variations in their protein sequences. In general, bacterial and fungal laccases share similar structural features, including the histidine-cysteine-histidine (His-Cys-His) configuration forming the copper T1 site (Fig. 2). In bacterial laccases, the fourth coordination position is occupied by methionine, whereas in fungal laccases, hydrophobic residues such as phenylalanine or leucine typically replace methionine (Kumar et al. 2021). These substitutions influence electron transfer efficiency and redox potential, thereby directly affecting substrate oxidation capacity and overall catalytic performance (Brugnari et al. 2021).

In this context, Kumar et al. (2021) analyzed structural diversity, redox potential, and secondary structure differences between fungal laccase isoenzymes from *Ganoderma lucidum* and recombinant bacterial laccases derived from various strains of *Yersinia enterocolitica*. According to the authors, differences in the secondary structure among isoenzymes play a key role in variations in catalytic potential and enzyme stability. Therefore, structural differences can lead to significant functional divergence, complicating direct comparisons of performance across studies.

Although microbial laccases exhibit broad biotechnological potential, many studies have focused on their application in environmental contexts, particularly in the treatment of textile wastewater. Effluents generated during dyeing processes are often characterized by high temperatures, wide pH ranges, and the presence of diverse chemical additives (Li et al. 2021; Rai et al. 2019). Under such conditions, most fungal laccases deactivate rapidly, whereas certain bacterial laccases can be applied directly due to their high tolerance to these stressors (Ahlawat et al. 2019). From an economic perspective, the ability of bacterial laccases to operate at elevated temperatures enables hot-water recycling, significantly reducing energy consumption during wastewater treatment (Li et al. 2022). Nevertheless, despite their superior stability, the application of bacterial laccases remains limited by their relatively low redox potential (~ 400 mV) (Ahlawat et al. 2019; Liu et al. 2021; Rahman et al. 2024), which restricts their efficiency toward highly recalcitrant aromatic compounds (Kontro et al. 2021) and highlights an unresolved trade-off between catalytic strength and operational durability.

## Recombinant expression of fungal vs. bacterial laccases

Regarding the recombinant expression of microbial laccases, as shown in Table 1, several hosts have been investigated. Among them, the yeast *Pichia pastoris* (*P. pastoris*) is the most widely used system for expressing fungal laccase genes (Wiśniewska et al. 2021). This methylotrophic yeast can increase protein expression by 10–1000-fold relative to native levels (Song et al. 2020), offering important advantages such as proper protein folding, post-translational modifications, and an efficient secretion system (Tülek et al. 2021a). Since *P. pastoris* lacks endogenous laccase genes, the recombinant proteins are secreted directly into the medium, simplifying detection without cell lysis (Li et al. 2021). However, it should be noted that *P. pastoris* is not a universal host, as some genes encoding specific isoforms are not successfully expressed even when derived from closely related fungi (Dao et al. 2021).

Other yeasts have also been explored. *Saccharomyces cerevisiae is* also considered a common eukaryotic host for laccase gene expression; however, its low yield and excessive glycosylation limit its industrial application (Asemoloye and Marchisio 2022). In comparison, *Aspergillus niger* expressed *Pleurotus ostreatus* laccase at yields 175 times higher than *S. cerevisiae* (Huang et al. 2024). Beyond *A. niger*, filamentous fungi represent attractive hosts due to their natural ability to secrete large amounts of extracellular proteins, perform complex post-translational modifications essential for enzyme stability, and, in some cases, their GRAS (Generally Recognized As Safe) status (Huang et al. 2024). Nonetheless, optimizing expression conditions, including medium composition and induction parameters, can further enhance yield and activity in these systems.

Despite these advances, many challenges have been reported in the expression of fungal laccases, which can impact enzyme efficiency and functionality. These include the presence of introns in the coding sequence, improper formation of disulfide bonds, and hyperglycosylation (Kesebir et al. 2021). Gene sequence editing to remove introns (Dao et al. 2021), the use of signal peptides to enhance secretion (Asemoloye and Marchisio 2022), and protein engineering to stabilize folding have been successfully applied to mitigate these drawbacks (Huang et al. 2024; Savinova et al. 2020).

In contrast, bacterial hosts offer advantages such as rapid growth, simpler cultivation, easier extraction, and tolerance to adverse conditions (Hajipour et al. 2020). *Escherichia coli* (*E. coli*) is the most common system for heterologous bacterial laccase gene expression (Table 2) due to its ease of handling (Chopra and Sondhi 2022). However, expression in *E. coli* often results in insoluble aggregates, intracellular accumulation, and formation of inclusion bodies, leading to low yields and difficult purification (Bian et al. 2022; van Eerde et al. 2022; Wang et al. 2019). To address these limitations, approaches such as co-expression with molecular chaperones (Huang et al. 2024) and the use of fusion tags (Kesebir et al. 2021) have been reported to enhance solubility and functional folding.

Beyond fungi and bacteria, plants have also been investigated as alternative hosts. van Eerde et al. (2022) successfully expressed the bacterial laccase Lac51 from *Pseudomonas putida* in *Nicotiana benthamiana*, with enzymatic properties and lignin-degradation potential comparable to those of the variant expressed in *E. coli*. Although plant-based systems can support post-translational modifications (van Eerde et al. 2022), current evidence remains limited to case-specific studies. Thus, plant expression systems should currently be regarded as an exploratory and complementary approach rather than an alternative to microbial platforms.

Beyond expression yield, recombinant production often results in differences in catalytic properties and stability compared to wild-type laccases (Kesebir et al. 2021; Wang et al. 2019). These variations arise from a combination of structural, biochemical, and cellular factors associated with heterologous expression systems. Recombinant laccases may undergo altered folding pathways or inefficient incorporation of copper ions into the T1, T2, and T3 centers, which directly affects electron transfer efficiency and redox potential (Ahlawat et al. 2019; Kumar et al. 2021).

Additionally, differences in post-translational modifications, particularly in glycosylation patterns, can impact enzyme stability and substrate accessibility (Asemoloye and Marchisio 2022; Huang et al. 2024; Zhuo et al. 2019). Moreover, genetic engineering strategies such as codon optimization, fusion tags, and mutagenesis may introduce subtle structural perturbations that impact catalytic efficiency or long-term stability (Tülek et al. 2021a; Xu et al. 2019). Collectively, these factors underscore that improvements in expression yield do not necessarily translate into enhanced catalytic performance or robustness at the process level.

This complexity is further reflected in the lack of a universally optimal heterologous host for laccase production. Despite advances in recombinant expression, the large-scale fermentation of laccases remains challenging due to host-dependent limitations, including low secretion efficiency, improper folding, proteolytic degradation, and variability in post-translational modifications (Huang et al. 2024; Kesebir et al. 2021). Although a wide range of hosts has been tested, no single system can be considered universally optimal for recombinant laccase production. Yeasts offer high expression and secretion capacities but face challenges with hyperglycosylation. Bacterial systems are inexpensive and fast but prone to aggregation, and plant systems remain largely experimental. Future studies should therefore prioritize systematic comparative analyses and targeted host engineering strategies to mitigate these bottlenecks and enable more predictable and industrially viable laccase biocatalysts.

## Culture media for recombinant laccase production

Laccase production can be optimized by selecting appropriate culture media and fermentation conditions. Agro-industrial residues can serve as accessible and sustainable substrates for this production, as demonstrated by Asemoloye and Marchisio (2022), Huy et al. (2021), Rahman et al. (2024), Suhaili et al. (2021), and Wahida et al. (2021). Wahida et al. (2021) reported that *P. pastoris* cultivated in 40% (v/v) Sago Bioethanol Liquid Waste (SBLW) showed recombinant laccase activity corresponding to 73% of that obtained with the standard buffered complex methanol medium (BMMH). Supplementation with yeast extract and glycerol further enhanced activity up to 2.1 times. However, higher SBLW concentrations reduced performance, likely due to the presence of toxic compounds. Similarly, Suhaili et al. (2021) observed that optimized SBLW supplemented with glycerol not only improved recombinant laccase activity but also enabled mediator-free dye decolorization (68.6% for Remazol Brilliant Blue R (RBBR). According to the characterization of SBLW, this residue is primarily composed of glycerol, glucose, and organic acids, which support biomass formation and enhance enzyme production, rather than acting as identified redox mediators. The original study did not investigate the presence of natural mediator compounds in SBLW; therefore, the observed mediator-free decolorization is more likely associated with increased laccase yield and activity under optimized cultivation conditions. These results highlight the potential of SBLW, which is often discarded into watercourses and can cause severe ecological impacts, as a viable and cost-effective culture medium, cheaper than the synthetic BMMH medium.

Other studies confirmed that medium optimization and cultivation parameters are decisive. For instance, Huy et al. (2021) found that the yeast extract peptone methanol (YPM) medium supported the highest rFolacc5 activity (8,860 U/L), while agitation above 210 rpm doubled yields compared to lower speeds. Asemoloye and Marchisio (2022) showed that organic nitrogen sources (malt, yeast extract, peptone) consistently outperformed synthetic ones for recombinant laccase expression in *S. cerevisiae.* Under optimized conditions for pH, temperature, and nitrogen and carbon sources, laccase activity in yeast strains was significantly higher than in non-optimized media.

In addition to carbon and nitrogen sources, the presence of metal cofactors in the culture medium is also a key factor influencing recombinant laccase production. Several studies report the addition of copper salts (typically CuSO₄) during laccase production for different purposes. For example, Cao et al. (2021) used copper supplementation to enhance catalytic activity and lignin utilization, indirectly supporting laccase function rather than increasing protein yield. In other studies, CuSO₄ is included in the culture medium to provide the essential cofactor for enzyme activity (Coria-Oriundo et al. 2021), or added during induction to promote proper folding, although its effect on expression levels is not specifically evaluated (Gupta et al. 2019). Overall, copper supplementation primarily affects enzyme maturation and catalytic activity, while its direct impact on recombinant protein yield remains poorly explored. Despite its essential role, copper may also exert toxic effects on cells (Cao et al. 2021).

Overall, current evidence indicates that while synthetic media provide reproducibility, agro-industrial residues and organic supplements offer promising sustainable and cost-effective alternatives, albeit with challenges in standardization and large-scale applicability.

## Catalytic efficiency and specificity - the role of chemical mediators

Despite the high potential of laccases, some isoforms may have difficulty oxidizing certain substrates due to their size or high redox potential. In this regard, enzymatic mediators, small molecules that can act as electron carriers, can facilitate substrate oxidation and increase catalytic efficiency and stability (Rahman et al. 2024). Some of the most used mediators in laccases include 2,2’-azinobis-3-ethylbenzothiazoline-6-sulfonic acid (ABTS), 2,6-Dimethoxyphenol (DMP), 2-Methoxyphenol (Guaiacol), 1-Hydroxybenzotriazole (HBT) (Rahman et al. 2024), syringaldazine (Wiśniewska et al. 2021) and 4-hydroxybenzaldehyde (Syringaldehyde) (Song et al. 2020).

In laccase–mediator systems, mediator concentration is a critical parameter that directly influences oxidation efficiency and process performance. Experimental studies have shown that mediator loading must be carefully optimized, as system efficiency depends not only on the nature of the mediator but also on its concentration and reaction conditions (Kontro et al. 2021; Li et al. 2021; Song et al. 2020; Wiśniewska et al. 2021). For instance, in the degradation of benzodiazepines, mediators such as HBT, ABTS, DMP, and vanillic acid were evaluated at concentrations of 0.5–2.5 mM, with a clear increase in removal efficiency at higher concentrations (Ostadhadi-Dehkordi et al. 2012). In practical applications, mediators are typically used at low millimolar concentrations, although this varies depending on the system and substrate. For example, concentrations around 0.1 mM have been reported in decolorization systems (Park et al. 2021), while higher concentrations in the range of 1–10 mM or 0.1–0.3 equivalents relative to the substrate may be required in organic oxidation reactions (Obleser et al. 2022).

Several studies highlight the efficiency of ABTS as a benchmark mediator. For instance, Song et al. (2020) showed that ABTS and DMP increased recombinant laccase activity by 2.56- and 1.39-fold, respectively, while guaiacol remained better oxidized by the native enzyme. Similarly, Li et al. (2021) reported > 90% phenolic degradation with ABTS, outperforming guaiacol and HBT. Wiśniewska et al. (2021) confirmed the high affinity of recombinant laccase KbLcc1 for syringaldazine, but noted lower activity with guaiacol, which reinforces its limited efficiency. Therefore, mediator effectiveness depends on substrate type, and reliance on mediators may not always improve the oxidation of all compounds.

Broader mediator screenings (Kontro et al. 2021) revealed that 2,2,6,6-Tetramethylpiperidine 1-oxyl (TEMPO) was the most effective in oxidizing veratryl alcohol, followed by syringyl derivatives, while ABTS and HBT ranked lower. Importantly, phenolic mediators are generally more environmentally friendly than N–OH types (HBT, VIO, HPI), which pose toxicity concerns (Cañas and Camarero 2010).

The cost of mediators is another important limitation for large-scale applications. Based on supplier data (from commercial supplier online catalogs such as Sigma-Aldrich/Merck), commonly used mediators exhibit a wide price range, for example: TEMPO (~ 31 USD g⁻¹), ABTS (~ 97.9 USD g⁻¹), violuric acid (~ 25 USD g⁻¹), and syringaldazine (~ 124 USD g⁻¹), while others such as HBT (~ 3.56 USD g⁻¹), guaiacol (~ 0.70 USD g⁻¹), and 2,6-dimethoxyphenol (~ 2.52 USD g⁻¹), are relatively less expensive. These variations further impact the economic feasibility of laccase–mediator systems, particularly at large scale.

Although mediators can enhance laccase activity, their use is often limited by toxicity and high cost, as several commonly used synthetic mediators raise environmental and handling concerns. In addition, the requirement for relatively high mediator concentrations and frequent replenishment increases process costs, making large-scale applications less feasible (Dahiya et al. 2023; Rahman et al. 2024). Overall, while mediators expand the applicability of recombinant laccases, future efforts should prioritize engineering laccases capable of efficient direct oxidation, reducing dependence on artificial mediators, minimizing toxicity, and improving cost-effectiveness for industrial applications.

### Engineering and optimization strategies for recombinant laccases

#### Expression vectors for recombinant laccase production

Expression vectors are essential tools for large-scale recombinant protein production, as they provide the genetic elements required for efficient gene cloning and expression in host organisms, including bacteria, yeasts, and plants (İncir and Kaplan 2024). These vectors typically include regulatory sequences such as promoters and terminators, which control transcription and translation of the target gene.

In the case of fungal laccases, the most widely used expression vector is pPICZαA, particularly in *P. pastoris* (Huy et al. 2021; Kontro et al. 2021; Li et al. 2022; Liu et al. 2019, 2020; Tülek et al. 2021a). This vector offers the advantage of an α-factor secretion signal, which facilitates extracellular release and simplifies downstream purification (Tülek et al. 2021a). For bacterial laccases, pET-series plasmids, such as pET28a (Ahlawat et al. 2019; Bian et al. 2022; Gupta et al. 2019; Liu et al. 2021; Zhang et al. 2024) and pET22b (Chang et al. 2022; Li et al. 2020, 2021, 2022; Tülek et al. 2021b; Wang et al. 2020), are most used, as they are designed for high-level expression in *E. coli*. However, these systems often face challenges with protein solubility (Dao et al. 2021), leading to the formation of inclusion bodies (Chopra and Sondhi 2022; Tülek et al. 2021a) and requiring the use of refolding strategies.

Inducible promoters are central to these vectors, allowing precise control over gene expression. Methanol-inducible promoters are widely applied in *P. pastoris* (Kontro et al. 2021; Li et al. 2021), while IPTG (isopropyl β-D-1-thiogalactopyranoside) is standard in *E. coli* systems (Ahlawat et al. 2019; Cheng et al. 2021). Alternative induction strategies, such as ultraviolet light (Chang et al. 2022), have also been explored. The choice of induction strategy has direct implications for scalability, cost, and operational safety, as methanol poses risks in large-scale fermentations (Bian et al. 2022). In contrast, IPTG, although effective, remains economically limited for industrial applications. Consequently, promoter and induction system selection should balance expression efficiency with process feasibility rather than maximizing yield alone.

## Codon optimization

Several strategies exist to increase recombinant laccase production levels. One of them is codon optimization, which enhances the expression of foreign genes by adapting their codon usage to the host’s translational machinery (Bian et al. 2022). This modification ensures that the gene sequence preferentially utilizes codons that are more efficiently recognized by the host organism, thereby improving translation efficiency.

In fungal systems, codon optimization has consistently improved laccase yields. For example, Tülek et al. (2021a) adapted the laccase gene and expression vector for *P. pastoris*, while Asemoloye and Marchisio (2022) optimized codons for *S. cerevisiae* to enhance *Trametes trogii* laccase production. Similarly, Sun et al. (2023) optimized *Trametes versicolor* laccase for *Aspergillus oryzae*, and Huang et al. (2024) described a 38.8% increase in enzymatic activity when the *Cerrena unicolor* laccase was codon-optimized for *A. niger*. In addition, Dao et al. (2021) obtained lower-than-expected protein production in *P. pastoris*, attributing the poor yield to the absence of codon optimization. These findings collectively indicate that codon adaptation is often decisive for efficient heterologous expression in yeasts and filamentous fungi.

Codon optimization has also been successfully applied to bacterial laccases. Cao et al. (2021) optimized the *Streptomyces coelicolor* laccase for *P. putida*, while Coria-Oriundo et al. (2021) and Bian et al. (2022) reported significant increases in protein production when optimizing *Streptomyces ipomoeae* and *Bacillus vallismortis* laccases for *E. coli*. These results reinforce that optimization is not restricted to eukaryotic systems but is equally relevant in prokaryotes.

Beyond codon usage, additional strategies can further enhance expression. For instance, Huang et al. (2024) demonstrated that co-expression of molecular chaperones (BIP1 and HacA) in *A. niger* improved folding efficiency and boosted laccase activity by 10.7% and 48.4%, respectively. This highlights the synergistic effect of combining codon optimization with folding assistance, ensuring not only higher expression levels but also improved protein quality.

In summary, codon optimization is a viable strategy to enhance recombinant laccase production, thereby improving expression efficiency in both fungal and bacterial systems. However, its success ultimately depends on the interplay with other factors, including host physiology, vector design, and protein folding mechanisms.

### His-tag

The addition of histidine tags (His-tags) is a widely used strategy to facilitate the purification and recovery of recombinant proteins. In laccase expression studies, the 6×His tag is the most commonly used configuration (Coria-Oriundo et al. 2021; Li et al. 2020; Liu et al. 2019; Mital et al. 2021; Xu et al. 2019; Zhang et al. 2024), while extended variants, such as 8×His, have also been reported (La et al. 2023). Although C-terminal fusion is conventional, successful N-terminal tagging has been demonstrated in *P. pastoris*, indicating that tag positioning can be flexible depending on the host and expression context (Tülek et al. 2021a).

His-tags enable straightforward purification using affinity chromatography. For instance, Chang et al. (2022) achieved 10.99 mg of purified LacMp1 in a single step, while other studies also obtained efficient recovery using HisTrap columns (Blánquez et al. 2019; Trubitsina et al. 2021). Alternative resins, such as HIS-Select^®^ gels, further expand purification options (Hajipour et al. 2020). Additionally, expression systems that encode His-tags, such as the pET SUMO One Shot vector, have been shown to enhance protein solubility and simplify downstream processing (Kesebir et al. 2021).

Interestingly, His-tags are not limited to purification. Wang et al. (2019) exploited their affinity for metal-organic frameworks (MOFs), immobilizing bacterial laccase on Fe₃O₄@ZIF-8 nanoparticles. This system maintained 75.7% activity after five cycles, highlighting the potential of His-tags for enzyme stabilization and reuse in biotechnological applications.

Overall, His-tags remain powerful tools for the purification and processing of recombinant laccase. Thus, their benefits must be weighed against potential impacts on enzyme structure, activity, and stability, particularly for applications requiring high catalytic efficiency or long-term operational performance (Chang et al. 2022).

### Mutagenesis

Protein engineering strategies, including mutagenesis, directed evolution, and ancestral sequence reconstruction (ASR), are valuable approaches. Among these, mutagenesis remains one of the most widely used strategies for improving microbial strains, particularly to increase recombinant protein production. Several studies have demonstrated its potential to improve enzyme yield and specificity; however, the effects can depend strongly on cofactors and the structural context. Two main approaches are commonly used: mutagenesis of the microbial host and direct mutagenesis of the laccase protein.

Using the Atmospheric and Room-Temperature Plasma (ARTP) technique, Huang et al. (2024) generated 20 mutant strains of *A. niger*, with one strain achieving a 68.6% increase in laccase yield compared with the parental strain, demonstrating the benefits of microbial strain mutagenesis for strain improvement. Gupta et al. (2019) explored random mutagenesis combined with heterologous expression of a laccase gene from *Rheinheimera* sp. They observed that certain mutations, such as premature stop codons, can abolish activity in the absence of cofactors while enhancing it in their presence, highlighting a trade-off between structural integrity and cofactor dependence. Savinova et al. (2020) reported that a mutant *Aspergillus nidulans* strain producing recombinant laccase from *Trametes hirsuta* showed improved steroid biotransformation, with progesterone conversion reaching 17.2% after 66 h, compared to 10.5% in control strains, suggesting that mutagenesis can expand laccase applications beyond dye decolorization.

Direct protein mutagenesis has also been widely applied to enhance enzyme performance. Yan et al. (2022) applied saturation mutagenesis to three residues in *Bacillus pumilus laccase*, achieving up to 3.15-fold improvement in Congo Red (CR) decolorization. One variant also showed enhanced structural stability due to hydrophobic substitutions, illustrating how mutagenesis can balance activity gains with stability changes.

In addition to these mutagenesis-based approaches, ancestral sequence reconstruction (ASR) has emerged as a powerful approach for laccase engineering. This method involves inferring and synthesizing ancestral enzyme sequences, which often exhibit enhanced stability, broader substrate scope, and improved expression (Barber-Zucker et al. 2022). Recent studies have shown that resurrected ancestral laccases can exhibit higher redox potentials and greater robustness than extant enzymes, making them attractive scaffolds for further engineering (Gomez-Fernandez et al. 2020). Furthermore, the combination of computer-guided mutagenesis and directed evolution has enabled significant improvements in redox potential, mediator activity, and overall catalytic performance (Barber-Zucker et al. 2022). Structural engineering strategies, such as modifying flexible surface loops, have also proven effective for enhancing thermostability without compromising activity (Mateljak and Alcalde 2021).

Overall, these strategies demonstrate that protein engineering can generate enzyme variants with improved catalytic efficiency, stability, and substrate specificity, while also highlighting potential trade-offs or cofactor dependencies associated with structural modifications.

### Computational insights into laccase structure and function

Computational approaches, such as molecular docking and molecular dynamics, are useful for understanding laccase structure, stability, and substrate specificity, complementing experimental studies. Dao et al. (2021) performed computational analyses of laccase isoforms from *Rigidoporus* FMD21, evaluating their interactions with chlorinated dioxins and standard substrates. Among the five natural isoenzymes, four displayed binding probabilities above 65% with 2,3,7,8-TCDD, reaching a maximum of 72%. Substrates such as ABTS, guaiacol, and 2,6-DMP generally showed good affinity, although two isoforms presented weaker interactions. These results aligned with experimental degradation assays, indicating that the efficiency of 2,3,7,8-TCDD removal is strongly dependent on isoenzyme-specific features.

Li et al. (2022) explored the molecular basis of thermophilicity in *B. pumilus* laccase through docking and molecular dynamics simulations. By testing the rLAC–ABTS complex at 325, 355, and 365 K, they observed that the RMSD decreased with increasing temperature, suggesting enhanced thermal stability. Complementary experiments confirmed these findings, showing decolorization rates above 70% for food dyes under optimal mediator, pH, and temperature conditions.

Additionally, Rawal and Suman (2024) employed homology modeling with MODELLER, docking, and 200 ns molecular dynamics simulations to investigate the interactions of recombinant laccases with chlorinated phenols. Their results highlighted key enzyme–substrate interactions, including hydrogen bonding and hydrophobic contacts, that underpin substrate specificity and structural stability and provide guidance for engineering more effective biocatalysts for environmental applications. Meanwhile, Huang et al. (2024) used AlphaFold2 to predict the three-dimensional structure of laccase Lac2 after removal of the signal peptide. The predicted model was used for molecular docking with six substrates, including textile dyes and fungal toxins, providing insights into enzyme–substrate interactions and structural determinants of specificity and stability.

In conclusion, homology modeling, molecular docking, and dynamics have provided important insights into the specificity and stability of recombinant laccases. When combined with experimental validation, these approaches support a better understanding of enzyme behavior and contribute to the development of more efficient biocatalysts for environmental applications.

### Applications of recombinant laccases in the environmental field

**Fig. 3:**
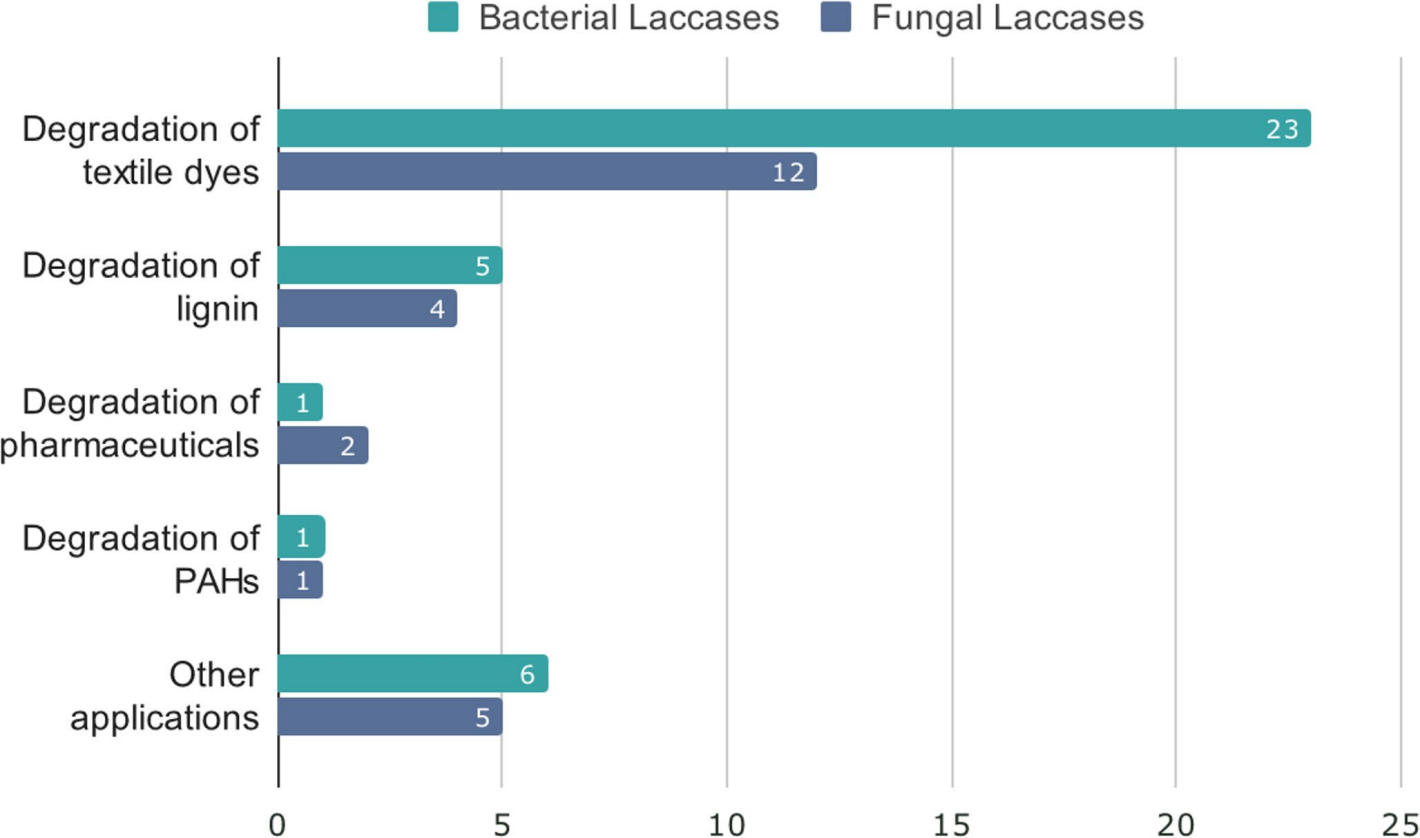
Comparison of fungal and bacterial recombinant laccase applications in reviewed articles from 2019 to 2025

Among the reviewed articles on recombinant laccases, 24 reported the heterologous expression of fungal-derived laccases, while 36 others focused on bacterial-derived laccases. Figure 3 compares the main reported applications of recombinant bacterial and fungal laccases (articles published from 2019 to 2025).

**Fig. 4:**
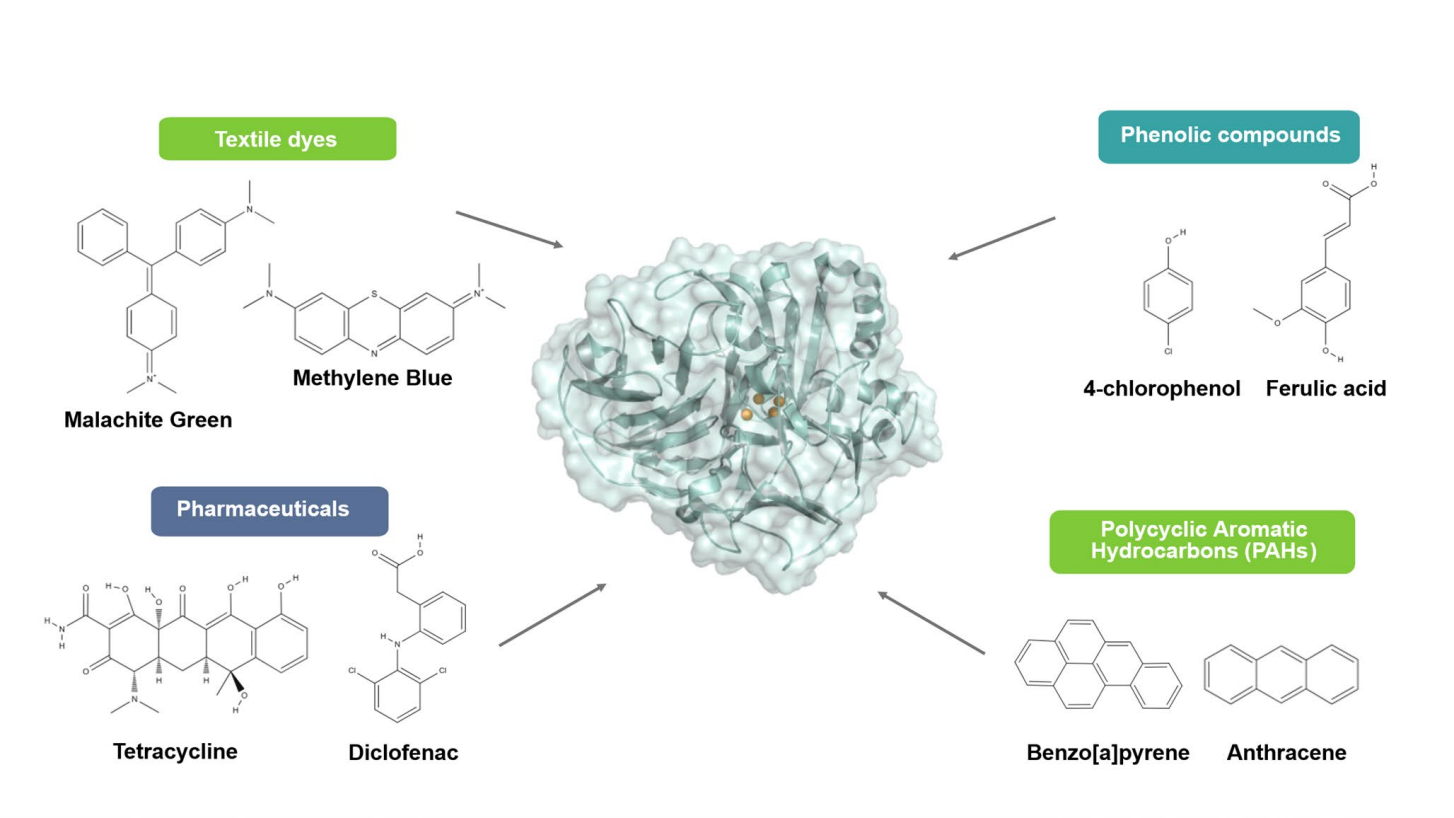
Representative substrates and environmental pollutants targeted by laccases

A schematic overview of representative substrates and environmentally relevant compounds oxidized by recombinant laccases is shown in Fig. 4. Specific parameters of recombinant fungal and bacterial laccases applications are detailed in Tables 1 and 2.

**Table 1:** Characteristics of different recombinant fungal laccases, including bibliographic reference, laccase synonym, main application, source organism, heterologous expression host, and expression vector used. It also details the optimal temperature for enzymatic activity and optimal pH of the enzyme, with the substrate used for each measurement provided in parentheses

Ref.	Synonym	Application	Native Source	Expression Host	Expression Vector	Optimal Temperature (°C)	Optimal pH
Asemoloye and Marchisio (2022)	Ttlcc1	Degradation of hydrocarbon	*T. trogii*	*S. cerevisiae* CEN.PK2–1 C	pRSII406 pRSII426	50	4.5 (ABTS) 5.0 (DMP) 5.5 (SGZ)
Aung et al. (2019)	LAC1	Degradation of textile dyes	*Aureobasidium melanogenum*	*A. melanogenum* (homologous)	pAPX13	40 (ABTS)	3.2 (ABTS)
Coria-Oriundo et al. (2021)	Lac2	Degradation of textile dyes	*Cerrena unicolor*	*A. niger* VT-2	pMD18	45 (ABTS) 55 (GUA)	4.0 (ABTS) 5.5 (GUA)
Dahiya et al. (2023)	LCC1-62	Degradation of textile dyes	*Cyathus bulleri*	*P. pastoris*	NR	NR	NR
Dao et al. (2021)	Rlac1-7	Degradation of dioxin	*Rigidoporus sp.* FMD21	*P. pastoris* X33	pPICZα B	NR	3.0/4.0 (ABTS) 4.0/5.0 (GUA) 3.0/4.0/5.0 (DMP)*
Huang et al. (2024)	lacA	Biotransformation of pharmaceuticals	*T. hirsuta* 072	*A. nidulans* 031	pBGlac	NR	NR
Huy et al. (2021)	FoLacc5	Degradation of textile dyes	*Fusarium oxysporum*	*P. pastoris* X33	pPICZαA	40 (ABTS)	3.5 (ABTS)
Jia et al. (2022)	Lac1	Degradation of textile dyes	*T. hirsuta* MX2	*P. pastoris* GS115	pPIC9K	60 (ABTS)	2.5 (ABTS)
Kontro et al. (2021)	CcLcc9	Degradation of lignin	*Coprinopsis cinerea*	*P. pastoris* X33	pPicZαA	50 (ABTS) 60 (DMP)	4.0 (ABTS) 7.0 (DMP)
La et al. (2023)	Lac1a	Degradation of lignin	*Hericium erinaceus*	*P. pastoris*	PPICZα	45 (ABTS)	3.5 (ABTS)
Li et al. (2022)	Lcc1	Degradation of phenols	*Coriolus versicolor*	*P. pastoris* KM71H	pPICZαA	40 (ABTS)	4.5 (ABTS)
Litwińska et al. (2019)	TVLCC5	Degradation of pharmaceuticals	*T. versicolor*	*Arxula adeninivorans* SBUG 724	Xplor3.2	50 (ABTS)	4.5 (ABTS) 5.0 (DMP) 5.5 (SGZ)
Liu et al. (2019)	Stlac2	Degradation of textile dyes	*Setosphearia turcica*	*P. pastoris*	PPICZαA	60 (ABTS)	4.5 (ABTS)
Liu et al. (2020)	LeLac	Degradation of rape straw	*Lentinula edodes*	*P. pastoris*	PPICZαA	60 (ABTS) 50 (OT)	3.0 (ABTS) 4.0 (OT)
Liu et al. (2021)	Lac-2	Degradation of lignin from corn straw	*P. ostreatus*	*P. pastoris* X33	pGAPZαA	50 (ABTS/DMP/GUA)	3.0 (ABTS/ DMP) 3.5 (GUA)
Otero et al. (2025)	Pnh_Lac1	Degradation of textile dyes	*Peniophora sp.* CBMAI 1063	*P. pastoris*	pGAPαA1	60 (ABTS)	3.0 (ABTS/GUA) 5.0 (SGZ)
Sabellico et al. (2025)	LCC1	Biodegradation of polymers	*T. trogii*	*Kluyveromyces lactis*	pDRLCi	NR	NR
Suhaili et al. (2021)	Lcc1	Degradation of textile dyes	*Marasmius cladophyllus*	*P. pastoris* GS115	Ppiczb	NR	NR
Sun et al. (2023)	TvLac 1–7	Degradation of polycyclic aromatic hydrocarbons	*T. versicolor*	*A. oryzae* Cols1300	NR	NR	NR
Tülek et al. (2021a)	Mmlac	Degradation of textile dyes	*Madurella mycetomatis*	*P. pastoris* X33	pPICZαA	50 (SGZ) 55 (ABTS) 60 (DMP)	4.0 (ABTS) 5.0 (SGZ/DMP)
Wang et al. (2024)	rLacF	Degradation of lignin from rice straw	*T. hirsuta* MX2	*P. pastoris* GS115	pPIC9K	60 (ABTS) 40 (DMP)	2.5 (ABTS) 4.0 (DMP)
Wiśniewska et al. (2021)	KbLcc1	Degradation of dyes and biotransformation	*Kabatiella bupleuri* G3 IBMiP	*P. pastoris*	pPINKα-HF	30 (ABTS)	3.5 (ABTS) 5.5 (GUA) 6.5 (SINAP) 7.0 (SGZ)
Xu et al. (2019)	Lcc9	Degradation of textile dyes	*Coprinopsis cinerea*	*P. pastoris* GS115	pPIC9K	70 (SGZ)	6.5 (SGZ)
Zhuo et al. (2019)	LACC6	Degradation of textile dyes	*Pleurotus ostreatus* HAUCC 162	*P. pastoris* GS115	pPIC3.5 K	NR	NR

*The pH values refer to the optimal activity range observed across different recombinant laccases (RLacs).*ABTS, 2,2′-azino-bis(3-ethylbenzothiazoline-6-sulphonic acid); DMP, 2,6-dimethoxyphenol; GUA, guaiacol; NR, not reported; OT, o-tolidine; SGZ, syringaldazine; SINAP, sinapic acid.*

**Table 2:** Characteristics of different recombinant bacterial laccases, including bibliographic reference, laccase synonym, main application, source organism, heterologous expression host, and expression vector used. It also details the optimal temperature for enzymatic activity and the enzyme’s optimal pH, with the substrate used for each measurement provided in parentheses

Ref.	Synonym	Application	Native Source	Expression Host	Expression Vector	Optimal Temperature (°C)	Optimal pH
Ahlawat et al. (2019)	yacK	Degradation of textile dyes	*Yersinia enterocolitica* (strain 7)	*E. coli* BL21	pET28a	70 (ABTS)	7.0 (ABTS)
Bian et al. (2022)	fmb-rL103	Mycotoxin degradation	*B. vallismortis* fmb-103	*E. coli* BL21 (DE3)	pET-28ª	80 (ABTS)	5,0 (ABTS)
Blánquez et al. (2019)	SilA	Degradation of textile dyes	*S. ipomoeae* CECT 3341	*E. coli* BL21 (DE3)	pET28	60 (ABTS)	NR
Bueno-Nieto et al. (2023)	FNTL	Degradação of polycyclic aromatic hydrocarbons (PAHs)	*Bacillus sp.* FNT	*E. coli* BL21	pJ444	70 (SGZ)	6.0 (SGZ)
Chang et al. (2022)	LacMp1	Degradation of textile dyes	*Marinomonas profundimaris*	*E. coli* BL21 (DE3)	pET-22b(+)	50 (ABTS/SGZ) 55 (K4Fe(CN)6/L-DOPA)	5.5 (ABTS) 7.0 (K_4_Fe(CN)_6)_ 7.5 (SGZ/l-dopamine)
Cheng et al. (2021)	CotA-laccase	Degradation of textile dyes	*Bacillus subtilis* ISW1214	*E. coli* BL21 (DE3)	pET22b	60 °C (ABTS)	5.0 (ABTS) 7.0 (CAT)
Chopra and Sondhi (2022)	rLacNS2324	Degradation of textile dyes	*B. licheniformis* NS2324	*E. coli* BL21 (DE3)	pet 15b	40 (GUA)	5.5 (ABTS) 6.0 (α-Napthol) 6.5 (SGZ) 8.0 (GUA) 10.0 (CAT)
Chopra et al. (2023)	rLacNS2324	Degradation of textile dyes	*B. licheniformis* NS2324	NR	pUC18	NR	NR
Coria-Oriundo et al. (2021)	SilA^I^ CotA^II^	Degradation of textile dyes	*S. ipomoeae* ^*I*^ *Bacillus subtilis* ^*II*^	*E. coli* BL21 (DE3*)*^*I*^ *P. pastoris* ^*II*^	pHISTEV30a^I^ pPICNHIS^II^	50 (DMP) ^I^ NR^II^	8.0 (DMP) ^I^ 8.0 (DMP) ^II^
Dai et al. (2021)	rlac1338^I^ lac2-9 ^II^ (mutant)	Degradation of textile dyes	*Marine microbial metagenome*	*E. coli* BL21(DE3)	pET-32a(+)	55 (ABTS) ^I^ 60 (ABTS) ^II^	6.0 (ABTS) ^I^ 6,5 (ABTS) ^II^
Diefenbach et al. (2024)	Lacc Pl L^I^ Lacc Ba L^II^	Petroleum hydrocarbon degradation	*Arvibaculum lavamentivorans* Pl L^I^ *Brevundimonas alba* Ba L^II^	*E. coli* BL21-Gold(DE3)	pET-26b(+)	40 (ABTS) ^I^ 50 (ABTS) ^II^	6.0 (ABTS/DMP) ^I^ 8.0 (ABTS/DMP) ^II^
Domínguez et al. (2021)	SilA	Degradation of lignin	*S. ipomoeae*	*E. coli* BL21	pET28A	45 (ABTS)	8.0 (ABTS)
Espina et al. (2021)	FNTL	Degradation of textile dyes	*Bacillus sp.* FNT	*E. coli* BL21	pJ444	70 (SGZ)	6.0 (SGZ) 4.0 (ABTS) 5.0 (GA)
Gupta et al. (2019)	RhLacc ∆ rhlacc	Degradation of textile dyes	*Rheinheimera sp.*	*E. coli* BL21 (DE3)	pET28a	55 (ABTS)	NR
Wang et al. (2020)	rLac	Degradation of textile dyes	*Bacillus amyloliquefaciens* TCCC 111,018	*E. coli* BL21 (DE3)	pET22b(+)	80 (ABTS/DMP)	5.5 (ABTS) 7.0 (DMP)
Hajipour et al. (2020)	NR	Degradation of textile dyes	*Bacillus subtilis*	*E. coli* BL21(DE3)	pET22b	50 (GUA)	6.6 (GUA)
Hsu et al. (2019)	yacK	Biotransformation of TNT	*Escherichia coli* K12	*E. coli* BL21 (λDE3)	pET21b	NR	4 0 (ABTS)
Liu et al. (2021)	rLac	Degradation of textile dyes	*B. pumilus* ZB1	*E. coli* BL21 (DE3)	pET28a(+)	80 (ABTS)	5.0 (ABTS)
Jiang et al. (2021)	BPLacc	Degradation of textile dyes	*B. pumilus* ARA	*E. coli* JM109	pET-20b	85 (ABTS) 75 (GUA)	3.5 (ABTS) 7.0 (GUA)
Kesebir et al. (2021)	CotA	Degradation of textile dyes	*B. licheniformis* O12	*E. coli* BL21 (DE3)	pET SUMO	92 (ABTS)	5.0 (ABTS)
Li et al. (2020)	rLac	Degradation of textile dyes	*Bacillus velezensis* TCCC 111,904	*E. coli* BL21 (DE3)	pET22b(+)	80 (ABTS/DMP)	5.5 (ABTS) 7.0 (DMP)
Li et al. (2021)	rLAC	Degradation of textile dyes	*Bacillus licheniformis* TCCC 111,219	*E. coli BL21* (DE3)	pET-22b (+)	80 (ABTS/DMP)	5.0 (ABTS) 7.0 (DMP)
Li et al. (2022)	rLAC	Degradation of food dyes	*B. pumilus* TCCC 11,568	*E. coli* BL21 (DE3)	pET-22b (+)	80 (ABTS)	6.0 (ABTS)
Maati et al. (2024)	rLac-HhC125	Biotransformation of organic compounds into coloured molecules	*Halalkalibacterium halodurans* C-125	*E. coli* BL21(DE3)	pET28-a(+)	50 (SGZ)	8.0 (SGZ)
Mustafa et al. (2024)	rILac	Degradation of textile dyes	*Bacillus megaterium*	*E. coli* BL21	pET21a	37 (GUA)	7.5 (GUA)
Nazar et al. (2022)	Blac	Degradation of rice straw lignin	*Bacillus ligniniphilus* L1	*E. coli* BL21	NR	NR	NR
Rai et al. (2019)	WSUCF1 laccase	Degradation of lignin from corn straw	*Geobacillus sp.* strain WSUCF1	*E. coli*	NR	NR	NR
Sánchez-SanMartín et al. (2024)	FNTL	Degradation of pharmaceutical	*Bacillus sp.* FNT	*E. coli* BL21	pJ444	70 (SGZ)	6.0 (SGZ)
Trubitsina et al. (2021)	CjSL	Degradation of textile dyes	*Catenuloplanes japonicus* VKM Ac-875	*E. coli* M15	pQE-30	70 (DMP/ABTS)	3.6 (ABTS) 9.2 (DMP)
Tülek et al. (2021b)	rLAC	Degradation of textile dyes	*B. licheniformis* TCCC 111,219	*E. coli* BL21 (DE3)	pET-22b (+)	80 (DMP/ABTS)	5.0 (ABTS) 7.0 (DMP)
van Eerde et al. (2022)	Lac51	Degradation of lignin	*Pseudomonas putida*	*Nicotiana benthamiana* (plant)	pEAQ- HT -DEST1	40 (SGZ)	7.0 (SGZ)
Wahida et al. (2021)	SLAC - Y230R (mutant)	Degradation of textile dye	*Streptomyces coelicolor*	*E. coli* BL21(DE3)	pET-23a (+)	85 (ABTS)	4.0 (ABTS)
Xiao et al. (2021)	BaCotA	Degradation of textile dyes	*Bacillus stratosphericus*	*E. coli* JM109	pQE30	70 (ABTS)	5.0 (ABTS)
Yadav et al. (2021)	rSLAC	Degradation of phenolic compounds	*S. coelicolor*	NR	NR	80 (DMP)	8.0 (DMP)
Yan et al. (2022)	CotA-laccase	Degradation of textile dyes	*Bacillus pumilus* W3	*E. coli* BL21(DE3)	pColdII	80 (ABTS-wild) 90 (ABTS-mutant)	2.6 (ABTS-both)
Zhang et al. (2024)	Lac3833	Degradation of lignin	*Bacillus cereus*	*E. coli* BL21 (DE3)	pET28a(+)	40 (ABTS/DMP/GUA)	4.5 (ABTS) 5.5 (DMP/GUA)

ABTS, 2,2′-azino-bis(3-ethylbenzothiazoline-6-sulphonic acid); CAT, catechol; DMP, 2,6-dimethoxyphenol; GA, gallic acid; GUA, guaiacol; NR, not reported; OT, o-tolidine; SGZ, syringaldazine; SINAP, sinapic acid.

### Textile Dyes

One of the most common applications of laccases is the decolorization of dyes in textile wastewater. Numerous studies (Tables 1 and 2) have investigated the efficiency of recombinant laccases relative to their wild-type isoforms, highlighting biotechnological advances to enhance their properties and applicability. Although recombinant laccases generally outperform their wild-type counterparts, their efficiency remains highly dependent on the dye structure, reaction conditions, and, in many cases, the addition of a mediator.

Recombinant fungal laccases have been widely explored due to their high catalytic activity; however, most systems require mediators to achieve high decolorization yields, which may limit their industrial scalability. For example, a laccase from *Aureobasidium melanogenum* achieved 25.7–84.1% decolorization across five dyes within 24 h (Aung et al. 2019), while *Kabatiella bupleuri* laccase efficiently decolorized Methylene Blue (MB) and Basic Fuchsin (BF), and was also applied to ferulic acid biotransformation (Wiśniewska et al. 2021).

Mediator-free systems remain comparatively rare, although exceptions exist, such as Stlac2 from *Setosphaeria turcica*, which decolorized 67% of Malachite Green (MG) in 15 min without mediators (Liu et al. 2019). Additionally, Dahiya et al. (2023) engineered LCC1-62 from *Cyathus bulleri*, achieving 60–95% decolorization of real textile effluents within 12 h without mediators, outperforming the wild-type enzyme.

Additional strategies have focused on improving enzyme robustness. A laccase gene from *C. cinerea* expressed in *P. pastoris* showed a threefold increase in specific activity and enhanced thermal and pH stability compared to the native enzyme (Xu et al. 2019). Likewise, immobilization in ZIF-8 nanocomposites significantly enhanced thermal stability and enabled multiple reuse cycles, achieving 9.3–11.8-fold higher stability than the free enzyme at 65 °C (Tülek et al. 2021b).

Substrate specificity remains a relevant limitation. The recombinant laccase rLac1 from *T. hirsuta* MX2 efficiently decolorized RBBR (92.6% in 3 h) but showed reduced activity toward other dyes. Although ABTS improved decolorization, as predicted by docking analyses, its thermostability was compromised relative to the native enzyme (Jia et al. 2022). Further optimization, combining codon optimization, mutagenesis, and chaperone co-expression, as demonstrated for *C. unicolor* laccase expressed in *A. niger*, resulted in high degradation of RBBR, indigo, and mycotoxins (Huang et al. 2024).

Bacterial laccases have also demonstrated considerable potential for dye decolorization (Table 2). Ahlawat et al. (2019) demonstrated the efficiency of recombinant laccases from *Y. enterocolitica* and *B. pumilus*, which decolorized Rose Bengal (RBG) (90.4%), MG (77.7%), and Congo Red (CR) (74.5%), in accordance with in silico molecular dynamics results. These enzymes showed not only high activity but also thermostability and tolerance to alkaline conditions, making them promising for large-scale applications.

The role of mediators in enhancing bacterial laccase performance has also been emphasized. Blánquez et al. (2019) tested the laccase from *S. ipomoeae*, which was ineffective against several dyes on its own. However, the addition of natural mediators such as syringaldehyde, methyl syringate, and acetosyringone increased decolorization by 4- to 7-fold. Similarly, Coria-Oriundo et al. (2021) introduced low-cost phenothiazine derivatives as mediators, enabling more than 80% decolorization of Indigo Carmine (IC) and MG within one hour, as well as substantial removal of azo and anthraquinone dyes. These findings highlight the importance of pairing laccases with suitable mediator systems to expand their substrate range.

Several studies have focused on laccases from *Bacillus* species, which consistently display robustness. Li et al. (2020) described that *Bacillus velezensis* laccase decolorized 42–94% of different azo, anthraquinone, and triphenylmethane dyes, but only when ABTS was added. Cheng et al. (2021) showed that *Bacillus subtilis* laccase achieved over 90% decolorization of MG in both freshwater and saline wastewater using acetosyringone as a mediator, without cytotoxic effects on fish cells. Jiang et al. (2021) purified a *B. pumilus* laccase that removed more than 90% of BRRB and 80% of Direct Black 19 (DB19) with mediators, showing exceptional stability under acidic to neutral conditions. In contrast, Liu et al. (2021) found that laccase expression in *B. pumilus* ZB1 was stress-induced and less efficient (16.43% of CR, 54.05% of CV, and 41.61% of RB), yet it still contributed to reducing cytotoxicity in CR hepatocytes.

Protein engineering and directed evolution have further expanded the potential of bacterial laccases. Dai et al. (2021) used error-prone PCR to generate a mutant marine laccase, rlac1338, which decolorized up to 90.5% of Acid Violet 7 (AV7) and 85% of Coomassie Brilliant Blue (CBB) in the presence of Ca²⁺ and ABTS, although activity against Amaranth was limited. Kesebir et al. (2021) optimized the expression of a *Bacillus licheniformis* laccase in *E. coli* using the PET-SUMO system, thereby preventing aggregation and facilitating purification. Although decolorization efficiency varied (51.2% for Acid Blue 1 but only 1.9% for CR), the enzyme maintained stability under acidic and alkaline conditions.

Innovative expression systems have also been explored to improve production and efficiency. Chang et al. (2022) expressed a marine bacterial laccase from *Marinomonas profundimaris* in *E. coli* using a light-inducible system, avoiding traditional inducers such as IPTG (Bian et al. 2022) or methanol (Song et al. 2020). This enzyme achieved nearly complete decolorization of IC (99.83%) and CR (99.54%), along with high removal rates for other dyes. These results demonstrate how tuning expression strategies can significantly enhance the applicability of recombinant laccase in dye degradation.

Finally, computational tools have proven valuable for predicting and validating bacterial laccase performance. Li et al. (2021) linked the thermostability and alkaline tolerance of *B. licheniformis* laccase to hydrogen bonding and salt bridges identified through molecular dynamics simulations, findings later confirmed by experimental assays with high decolorization efficiencies (RB19 and RB5 dyes were decolorized by 95% and 90%, respectively). Likewise, Chopra et al. (2023) combined docking simulations with experimental tests, showing that *B. licheniformis* laccase had strong binding affinities for leather dyes such as Acid Orange 56 (AO56), Acid Red 97 (AR97), and Acid Yellow 110 (AY110), correlating with decolorization rates above 90% without mediators. Overall, these studies demonstrate how in silico and experimental approaches complement each other in guiding enzyme engineering and its applications.

Therefore, both fungal and bacterial laccases exhibit significant potential for decolorizing various dye classes, particularly when used in conjunction with mediators. Fungal laccases stand out for their high catalytic activity and broad applications, while bacterial laccases offer additional advantages, such as greater stability under extreme pH, temperature, and salinity conditions. These findings highlight the potential of laccases as sustainable and effective tools for treating industrial and textile wastewater (Table 3). 

**Table 3:** Comparative analysis of recombinant laccases performance in dye decolorization

Fungal Laccases															
Ref.	Synonym			Decolorization efficiency		Time (h)		pH		T (°C)		Medi-ator		Additional observations	
Dahiya et al. (2023)	LCC1-62			Disperse Red (95%)		12		4.0		30		None		Wild-type laccase achieved less than 40% decolorization.	
Huang et al. (2024)	rLac2			RBBR (91.64%), IB (87.15%), and AR1 (51.34%) in 24 h		24		4.5		45		None		NR	
Huy et al. (2021)	rFoLacc5			Aniline Blue (90.87%), Methyl Orange (82.80%), RBBR (79.61%), IC (73.44%)		24		NR		NR		None		Efficient degradation without mediators.	
Jia et al. (2022)	rLac1			RBBR (99.2%), AR1 (67.1%), CV (38.9%), NR (52.3%)		3		5.0		28		ABTS		Without ABTS: RBBR (92.57%), AR1 (67.1%), CV (14.2%), NR (12.3%).	
Liu et al. (2019)	Stlac2			MG (67.08%)		0.25		4.5		60		None		Unable to oxidize ABTS or 2,6-DMP	
Suhaili et al. (2021)	Lcc1			RBBR (68.6%)		360		NR		24		None		Cultivated in sago bioethanol liquid waste.	
Tülek et al. (2021a)	Mmlac			Amido Black 10B (~ 62%), Remazol Brilliant Yellow (58%), Rhodamine 6 G (43%), and CV (33%)		120		4.0		25		ABTS		NR	
Xu et al. (2019)	rLcc9			IC (99.1%; 100 min), 6B (98.6%, 80 min), K7R (94.9%; 140 min), KD8B (89.2%; 1 h), KM8B (82.5%; 1 h), M2GE (71%; 120 min)		-		6.5		30		MS		3x higher specific activity than wild-type; improved thermal and pH stability.	
Zhuo et al. (2019)	LACC6			MG (91.5%), RBBR (84.9%), BB (79.1%), MO (73.1%)		24		5.0		25		None		Three isoforms were tested: LACC6 decolorized most effectively.	
**Bacterial Laccases**															
Ahlawat et al. (2019)		Lacc DSKK1	RBG (90.4%), MG (77.7%), CR (74.5%)		16 h		8.0		37		None		Lacc *Y. enterocolitica* strain 7: MG (70.45%), RB (50.29%), CR (13.96%).		
Blánquez et al. (2019)		SilA	RB5 (29.34%), IC (27.32%), OII (14.2%), CRR (12.05%), TART (9.24%), AZB (0%)		24 h		8.0		35		None		The addition of SA, MeS, and AS resulted in a 4-, 6-, and 7-fold increase in decolorization, respectively.		
Chang et al. (2022)		LacMp1	IC (99.83%), CR (99.54%), Eriochrome black T (88.41%), RB 4 (51.61%)		24		7.0		30		None		NR		
Cheng et al. (2021)		CotA-laccase	MG (90% in freshwater and 93% in seawater) in 24 h.		24 h		5.0		37		AS		Reduced decolorization in tilapia pond wastewater, possibly due to unknown chemicals or bacteria interfering with MG degradation.		
Chopra and Sondhi (2022)		rLacNS2324	MB (99.28%), BPB (85.55%), Phenol Red (75.53%), Carbol Fuchshin (67.60%), Safranin (65.74%), CV (56.04%), Eosin Yellow (38.01%)		4		8.0		40		None		NR		
Dai et al. (2021)		lac2-9	AV7 (90.5%), BPB (67.8%), CBB (85%) and AMP (14.5%).		24		6.5		37		ABTS Ca²⁺		Laccases alone exhibited no degradation activity.		
Espina et al. (2021)		FNTL	FNTL was able to decolorize 8 different synthetic dyes (CR, MO, MR, CBBR250, BRB, MG, CV, RBBR).		6		6.0		40		AS		Partial decolorization of MG and MR without a mediator.		
Gupta et al. (2019)		∆RhLacc	IC (44.3%)		1 h		8.0		55		CuSO_4_		Without CuSO4: IC (0%)		
Wang et al. (2020)		rLAC	< 30% decolorization alone of AZP, CR, ABB, RB5, RB19, RBBR, CR, IC, MG, but 38–95% with mediators (ABTS, AS and SA) across pH 5.5–9.0, with highest at pH 5.5 (ABTS) and 7.0 (AS/SA).		6		5.5 7.0		60		ABTS, AS SA		NR		
Hajipour et al. (2020)		Laccase from *B. subtilis*	BB (54.9%), RBO3R (47.15%), RR106 (46.16%), and TBHF6 (30%).		6 h		6.0		50		CuSO_4_		NR		
Huang et al. (2024)		SilA	MG (93%) in 3 h (pH 8.0/24°C). XYL (86%) after 5 h (pH 8.0, 50 °C). RBBR (50%) after 24 h (pH 8.0, 24 °C).		-		-		-		PhCOOH		Effective in tap water. CotA is less productive but has a similar profile.		
Jiang et al. (2021)		BPLacc	RBBR (> 90%) with ABTS at pH 4.0/7.9; Direct Black 19 > 80% at pH 4.0 with any mediator (32.6% without mediator); Acid Red A35 and Acid Black ATT < 60% even with mediators.		4		4.0 7.9		50		ABTS GUA HBT VA AS		NR		
Kesebir et al. (2021)		CotA	acid black 1 (51.2%), methylene blue (36.2%), reactive black 5 (32.05%), Acid Red 27 (21.24%), Orange (13.1%), and CR (1.9%).		2		NR		NR		None		NR		
Li et al. (2020)		rLAC	42%−94% (AZP, CR, ABB, RB5, RB19, CV, IC, MG).		6 h		5.5–9.0.5.0		60		ABTS		Absence of mediators: <27% to all dyes.		
Li et al. (2021)		rLAC	AZP (100% with AS/SA, pH 7.0); ABB (100% with AS/SA, pH 7.0); RB19 (95% with ABTS, pH 7.0); RB5 (90% with ABTS, pH 9.0); CV (100% with AS, pH 7.0); IC (100% with ABTS pH 7.0, AS pH 7.0 and 9.0, SA pH 5.0 and 7.0)		6		5.0 7.0 9.0		60		ABTS AS SA		NR		
Liu et al. (2021)		rLac	CV (54.05%), RB4 (41.61%), and CR (16.43%).		120		NR		37		None		NR		
Trubitsina et al. (2021)		CjSL	BG (94%, pH 4.0), MO (57%, pH 4.0), MR (46%, pH 6.5), CR (31%, pH 4.0), MG (22%, pH 4.0).		24		4.0 6.5		30		ABTS		The purified laccase alone was able to efficiently decolorize only the azo dye CR (36%).		
Yan et al. (2022)		CotA- mutant T377I/T418G^I^ CotA- mutant G323S/T377I /T418G^II^	CR (76.59%^I^ and 59.37%^II^)		24		8.0		50		None		Mutants exhibited decolorization activity 3.15 and 2.44 times higher than the wild-type enzyme, respectively.		

*6B, Acid Red 6B; ABB, Adizol Black B; ABTS, 2,2′-Azino-bis(3-ethylbenzthiazoline-6-sulfonic acid); AMT, Amaranth; AR1, Acid Red 1; AS, Acetosyringone; AV7, Acid Violet 7; AZB, Azure B; AZP, Azophloxine; BB, Brilliant Blue; BG, Brilliant Green; BPB, Bromophenol Blue; CBB, Coomassie Brilliant Blue; CBBR250, Coomassie Brilliant Blue R250; CR, Congo Red; CRR, Cresol Red; CV, Crystal Violet; EBT, Eriochrome Black T; HBT, 1-Hydroxybenzotriazole; IB, Indigo Blue; IC, Indigo Carmine; K7R, Reactive Brilliant Orange K-7R; KD8B, Reactive Brilliant Red KD-8B; KM8B, Reactive Brilliant Red KM-8B; M2GE, Reactive Blue M-2GE; MB, Methylene Blue; MG, Malachite Green; MeS, Methyl Syringate; MO, Methyl Orange; MR, Methyl Red; MS, Methyl Syringate; NR, Neutral Red; OII, Orange II; PhCN, β-(10-Phenothiazyl)-propionitrile; PhCOOH, β-(10-Phenothiazyl)-propionic acid; PhZ, Phenothiazine; RB19, Reactive Blue 19; RB5, Reactive Black 5; RB5-Blue, Reactive Blue 5; RBBR, Remazol Brilliant Blue R; RBG, Rose Bengal; RBO3R, Remazol Brilliant Orange 3R; RR106, Remazol Red 106; SA, Syringaldehyde; TART, Tartrazine; TBHF6, Turquoise Blue HF6; VA, 5-Hydroxyiminobarbituric acid; XYL, Xylidine Ponceau*

### Agroindustrial waste and lignin management

Lignin, a primary component of lignocellulosic biomass, poses a significant challenge in the valorization of agroindustrial wastes. In this context, laccases emerge as promising enzymes for lignin degradation and removal, as well as for transforming lignocellulosic waste into higher-value products.

Several studies have explored the application of recombinant fungal laccases in different types of agroindustrial residues. For instance, Niglio et al. (2019) reported that the recombinant laccase poxa1b promoted partial delignification of apple pomace in a bubble column reactor, depolymerizing lignin chains into soluble products and improving cellulose accessibility. Similarly, Song et al. (2020) demonstrated the efficiency of a recombinant *P. ostreatus* laccase in degrading lignin from corn straw. Compared to the native enzyme, the recombinant laccase exhibited higher thermal stability, better activity under acidic conditions, and a greater degradation capacity (18.36% vs. 14.05%).

Further applications have been described in agricultural residues. Liu et al. (2020) expressed a *Lentinula edodes* laccase gene in *P. pastoris* and applied the recombinant enzyme in rapeseed straw. The recombinant enzyme efficiently degraded lignin at pH 4.0, reducing its content to 21.3% and enhancing cellulose hydrolysis by cellulases. In addition, soluble phenols were effectively removed, thereby improving enzymatic hydrolysis and reducing sugar losses. Wang et al. (2024) investigated the use of a recombinant *T. hirsuta* MX2 laccase (rLacF) in rice straw degradation. This enzyme showed higher thermal stability compared to other fungal laccases and was most effective in a stepwise hydrolysis strategy (cellulase → laccase → hemicellulase), resulting in a 39% increase in reducing sugar yield compared to inactivated laccase.

Recombinant laccases have also been applied to lignin derived from wood refining processes. Kontro et al. (2021) tested rCcLcc9 from *C. cinerea* for the depolymerization of hardwood lignin. In the presence of phenolic mediators such as syringyl nitrile and syringate, lignin underwent substantial structural modifications, leading to the production of value-added aromatic compounds, including vanillin, vanillic acid, syringaldehyde, syringic acid, and p-hydroxybenzoic acid. These findings highlight the potential of rCcLcc9 as a biocatalyst for lignin valorization into industrially relevant products.

In addition to fungal enzymes, bacterial laccases have gained attention for lignin valorization. Cao et al. (2021) further optimized secretion devices for *S. coelicolor* laccase in *P. putida*, demonstrating that the free-cell system was 13 times more effective in lignin utilization than immobilized complexes. Rai et al. (2019) employed recombinant laccases from the genus *Geobacillus* for the pretreatment of corn straw and bagasse. When combined with commercial enzymes (Accellerase 1500 and Cellic CTec2), the recombinant bacterial laccase significantly enhanced hydrolysis efficiency, resulting in a 2.28-fold increase compared to using commercial enzymes alone. Similarly, Domínguez et al. (2021) applied *S. ipomoeae* laccase to functionalize Kraft lignin, yielding stable, biodegradable oleogels with reactive properties. Nazar et al. (2022) expressed a laccase gene from *Bacillus ligniniphilus* and demonstrated that the resulting enzyme, when applied to rice straw, reduced lignin content by 8.93% and phenolic compounds by 44.8%, supporting bioethanol production in an ecologically sustainable way.

Therefore, recombinant fungal and bacterial laccases are powerful tools for the valorization of agroindustrial waste. By improving lignin degradation, enhancing cellulose accessibility, and promoting sugar release, they contribute to more efficient biomass conversion. Moreover, their application aligns with the development of biofuels and other bioproducts, reinforcing the role of recombinant laccases in advancing a circular and sustainable bioeconomy.

### Biodegradation of chemical and pharmaceutical compounds

Pharmaceutical residues are a significant source of environmental contamination, as many drugs are poorly absorbed by the human body and are excreted in their active form. Combined with the improper disposal of unused or expired medications, these substances reach aquatic environments, where they pose risks to ecosystems and public health (Litwińska et al. 2019).

Recombinant laccases have emerged as promising tools to address this challenge. For instance, Litwińska et al. (2019) cloned laccase genes from *T. versicolor* and expressed them in *Arxula (Blastobotrys) adeninivorans*, chosen as the host due to its cytochrome P450 system, which could complement pharmaceutical degradation. Among the laccases tested, only Tvlcc5 was successfully expressed on the surface of the yeast cell. In the presence of the mediator ABTS, Tvlcc5 completely degraded diclofenac and sulfamethoxazole within 1 h, whereas carbamazepine remained resistant. Similarly, Sánchez-SanMartín et al. (2024) expressed a bacterial laccase gene from *Bacillus* sp. FNT, showing that in the presence of acetosyringone, tetracycline degradation reached 91% in 24 h, without ecotoxic effects. These findings underscore the potential of recombinant laccases in pharmaceutical wastewater treatment, while also revealing substrate-specific limitations.

Beyond pharmaceuticals, recombinant laccases have been investigated for the degradation of endocrine disruptors and steroids. Li et al. (2021) described that *Coriolus versicolor* laccase, in combination with ABTS, degraded over 90% of 4-tert-octylphenol and octylphenol within 12 h, compared to less than 50% degradation without a mediator.

Recombinant laccases have also been explored for the degradation of hydrocarbons and polycyclic aromatic hydrocarbons (PAHs). Asemoloye and Marchisio (2022) expressed *T. trogii* laccase genes in *S. cerevisiae* strains, with the integrative plasmid variant (byMM935) achieving 98% degradation of total petroleum hydrocarbons with CuSO₄. Similarly, Sun et al. (2023) expressed seven laccase gene homologs from *T. versicolor*, among which TvLac5 showed 9.6 times higher activity against syringaldazine (pH 5) compared to a commercial laccase. On the other hand, Bueno-Nieto et al. (2023) demonstrated that *Bacillus* sp. FNT laccase oxidized anthracene and benzo[a]anthracene directly, while benzo[a]pyrene required mediators. Diefenbach et al. (2024) further reported that laccase from *Parvibaculum lavamentivorans* reduced total petroleum hydrocarbons in soil by 83% (w/w), outperforming isoforms from *Brevundimonas alba* and *Pseudoxanthomonas mexicana*.

Applications have also extended to polymer degradation. Sabellico et al. (2025) demonstrated that *T. trogii* laccase (LCC1), expressed in *Kluyveromyces lactis* and combined with ABTS or DMP, promoted the oxidative degradation of UV-exposed polypropylene by inserting oxygenated groups into the polymer chain, thereby facilitating further biodegradation. Similarly, Zhang et al. (2024) cloned laccase genes from *Bacillus cereus* and *Ochrobactrum pseudintermedium* (buffalo rumen isolates), showing sodium lignosulfonate degradation efficiencies of 3.77% (enzyme alone) and 8.39% (enzyme + mediator). Another relevant application of laccases is in the degradation of toxic compounds such as mycotoxins and nitroaromatic pollutants. Bian et al. (2022) demonstrated that recombinant laccases degraded over 50% of aflatoxin B1, a potent human carcinogen, within 48 h, underscoring their potential for food safety applications.

Recombinant laccases have been explored for the biotransformation of various chemical compounds (Hsu et al. 2019; Savinova et al. 2020; Wiśniewska et al. 2021; Xiao et al. 2021). For instance, Hsu et al. (2019) expressed the bacterial laccase gene yacK in *E. coli* to transform TNT, a highly toxic and persistent nitroaromatic compound; while laccases can detoxify pollutants, the resulting metabolites were even more toxic, highlighting potential risks in enzymatic degradation pathways. Similarly, Savinova et al. (2020) used *A. nidulans* expressing *T. hirsuta* laccase to convert progesterone into steroidal precursors with pharmaceutical value. These studies demonstrate that, although laccases possess significant biotransformation potential, a careful evaluation of both products and pathways is crucial to ensure safe and effective applications.

Finally, strategies to improve catalytic performance and reusability have been widely investigated. Yadav et al. (2021) immobilized *S. coelicolor* laccase on magnetic nanoparticles, achieving ≥ 98% degradation of phenolic compounds (phenol, 4-chlorophenol, and 4-fluorophenol) within 2 h with a reduction in toxicity of up to 90%. The immobilized enzyme also exhibited enhanced stability and reusability. Enzyme immobilization, as noted by several authors (Tülek et al. 2021b; Wang et al. 2019; Yadav et al. 2021), offers several advantages, including improved chemical and thermal stability, extended storage life, enhanced catalytic efficiency, and the potential for enzyme recovery and reuse.

### Limitations of the use of recombinant laccases in environmental applications

Several challenges have been reported that limit the application of recombinant laccases in environmental fields. These include (I) the lack of commercially available laccase products specifically developed for pollutant degradation, (II) challenging environmental factors such as inhibitors, temperature, and a wide pH range, which negatively impact the stability and efficacy of enzymes in real water and soil environments (Sun et al. 2023), as well as (III) the high cost of production and application, mainly due to the use of expensive synthetic fermentation media and limited access to mediators (Singh and Arya 2019).

Therefore, feasibility studies are needed to demonstrate the cost-benefit of using laccases for pollutant removal. Additionally, employing synthetic biology techniques to modify laccases, as reported in some studies (Gupta et al. 2019; Huang et al. 2024; Savinova et al. 2020; Yan et al. 2022), may enhance their stability and activity under adverse conditions.

Moreover, among the popular strategies to reduce production costs is the adoption of alternative fermentation raw materials, with agricultural and industrial waste becoming preferred options (Suhaili et al., 2021).

In addition, several studies have observed inconsistencies in the molecular weight of recombinant laccases. Typically, after heterologous expression, recombinant laccases often present a higher apparent molecular weight. This can be explained by post-translational modifications that occur in eukaryotic expression systems, such as glycosylation (Tülek et al. 2021a). While glycosylation is important for the enzyme’s function, at high concentrations, it can form aggregates, reducing the enzyme’s activity in pollutant degradation processes, for instance. It can also pose a barrier to efficient heterologous expression (Liu et al. 2019).

These inconsistencies have primarily been observed in the expression of fungal laccases, as seen in the study by Huy et al. (2021), where the predicted size of rFoLacc5 was 69 kDa, whereas SDS-PAGE and western blot analysis indicated that the enzyme was ≥ 100 kDa. Litwińska et al. (2019) reported the same issue, with Tvlcc5 having a molecular weight of 100 kDa, significantly higher than the predicted 53.6 kDa based on the amino acid sequence. In Liu et al. (2019), the predicted molecular weight of Stlac2 was 61.64 kDa; however, upon expression, the resulting protein showed an apparent molecular weight of approximately 100 kDa. The authors observed that the recombinant protein produced in *P. pastoris* was inactive because a potential N-glycosylation site blocked the necessary water exit for catalytic oxidation. In other studies, differences were also found, but not exceeding 20 kDa, as observed in (Jia et al. 2022; Wiśniewska et al. 2021; Zhuo et al. 2019).

In addition to academic research on recombinant laccases, biotechnology companies also offer commercial products primarily for basic research and biocatalysis. Examples include fungal laccases from *T. versicolor* (Cusabio) and *Aspergillus sp.* (Sigma-Aldrich), as well as a recombinant laccase from *B. subtilis* (Creative Enzymes) and other microbial laccases provided by Kerafast, MyBioSource, and SwissAustral. These products are typically supplied in purified form and exhibit well-characterized in vitro activity. However, their relatively high cost, combined with the need for specific conditions to achieve optimal activity, remains a barrier to their large-scale application in wastewater treatment or the degradation of persistent organic pollutants.

### Final considerations

Recombinant fungal and bacterial laccases have emerged as promising biocatalysts to meet the growing demand for efficient, sustainable enzymes in environmental and industrial applications. Beyond improved production yields, the advantages frequently reported for recombinant laccases in terms of activity and stability can be mechanistically attributed to controlled heterologous expression strategies, including optimized folding, tailored post-translational modifications, improved copper incorporation, and the rational introduction of stabilizing mutations. In this context, the combination of codon optimization, fusion tags, mutagenesis, and in silico analyses enables the deliberate tuning of laccase properties rather than simply reproducing native enzymes.

At the same time, these benefits depend strongly on the choice of expression host, fermentation conditions, and downstream processing, which may also introduce trade-offs, such as mediator dependence or variability in catalytic efficiency. Advances in expression vector design, fermentation optimization, and the use of low-cost substrates, including agro-industrial residues, represent viable strategies to improve productivity and economic feasibility. In addition, the cost of redox mediators remains a critical factor that may limit large-scale applicability, highlighting the need to develop more cost-effective mediator systems or mediator-free approaches. In parallel, emerging approaches based on AI-aided rational enzyme design and high-throughput screening are expected to accelerate the identification of robust laccase variants. Meanwhile, the genetic and metabolic engineering of fungal host cells offers additional opportunities to enhance secretion efficiency and facilitate large-scale production.

Despite remaining challenges, recombinant laccases demonstrate significant potential for sustainable applications, including textile dye degradation, lignin valorization, and the removal of pharmaceuticals and other toxic pollutants. The successful translation of these enzymes into real-world environmental remediation systems will require integrating enzyme engineering, low-cost production strategies, and process-level solutions, supported by feasibility and techno-economic analyses. Future research should further address key operational and economic aspects, including enzyme production costs, stability under industrial conditions, lifetime, recovery, and reuse, as well as scalability constraints associated with different expression systems. Although a comprehensive techno-economic evaluation is beyond the scope of this review, these factors are essential to bridge the gap between laboratory-scale studies and industrial implementation. Such an integrated framework is essential to consolidate the commercial and environmental relevance of recombinant laccases in emerging green technologies.

## Data Availability

No datasets were generated or analysed during the current study.

## Declarations

### AI

The authors used ChatGPT (OpenAI) solely to assist with language editing and to improve the clarity of the manuscript. All scientific content, interpretation, and conclusions are the sole responsibility of the authors.
